# The “Brain to Behavior Approach” to diagnosis and treatment

**DOI:** 10.3325/cmj.2015.56.500

**Published:** 2015-10

**Authors:** Lukas M. Konopka

**Affiliations:** Spectrum Center for Integrative Neuroscience, McHenry, IL, USA *lmkonopka@gmail.com*

Sadly, the Decade of the Brain has passed, an exciting time that seemed to promise the long awaited identification of psychiatric biological markers (1). Despite the significant advances in brain imaging and biochemical techniques such as genetic mapping, we failed to achieve greater accuracy in our diagnostics or more personalized care for patients with psychiatric conditions. Even now, we search for that Holy Grail of mental health, a specifically sensitive clinical biological marker for psychiatric diagnosis, and as we have continued to search, we have discovered that the mental health profession struggles to think outside its box. Perhaps our persistent avoidance of empirical data has constrained our creativity, making it difficult for the various theoretical silos to reflect upon their clinical weaknesses. Historically, we have convinced ourselves that we can know and diagnose our patients simply by scrutinizing their symptoms and correlating them with specific underlying biological disturbances. Although this may be true when a specific well-defined brain lesion involves specific sensory systems, for example when blindness results from a visual pathway lesion, it may be quite uncertain when frontal lobe or basal ganglia lesions silently interfere with mood and cognition without disturbing specific motor performance (2). As such, when we search for the unique biological marker for a single psychiatric diagnostic category, we set ourselves up for failure. Despite research eventually discrediting all initial leads that promised to find a particular diagnostic marker, their efforts were not in vain. Today we have re-evaluated certain measures only to discover new utilities. Current examples of such rediscovered tools are quantitative EEG, which despite its less than stellar beginnings is showing clear clinical applications in psychiatric diagnosis (3,4) and cortisol, which now appears as a marker for depression (5) and, perhaps, posttraumatic stress disorder (6-10).

Today, psychiatry’s and psychology’s standards of care generally use a single clinical interview to determine a patient’s diagnosis and initiate treatment. For psychiatry, medication trials are the treatment of choice and yield highly variable results. For psychology, treatments are determined by a therapist’s education and expertise, making treatments' effectiveness difficult to evaluate because they often lack objective outcome measures as part of their standards of practice (11). We commonly understand mental health as independent of the therapeutic method and the relationship between the practitioner and patient as the key to a successful therapeutic outcome (12). Moreover, data strongly support the idea that combining pharmacological treatment with psychological therapy is more important than either one (13). But, we do better! Once when we lacked available, objective, reproducible, assessment data for characterizing our patients, we were justified in basing our treatments on symptomatology. (In fact, I have personally struggled with the same diagnostic and therapeutic issues.) Nevertheless, I found that even when we use scrupulous assessment to classify patients by their presenting symptoms, we repeatedly discover that a common diagnosis has highly variable biological underpinnings. Therefore, I suspect that patients with similar symptoms, within a general diagnostic category, have difficulties caused by significantly variable biological substrates. Surprisingly, when we evaluate specific differences using physiologic measures, we find significant variability between individuals within a diagnostic group. For example, a study using clinically similar PTSD patients showed that when we divided the patients by their auditory intensity patterns, they fell into two clinically distinct subpopulations (14). In another study, we found that patients, who were cocaine-preferring abusers, also fell within defined subgroups (15). In addition, we could define patients who responded to electroconvulsive therapy based on their brain perfusion results (16). Such studies led me to focus on individual patients using objective imaging and behavioral measures to help me understand their clinical presentations. My experience with this approach led me to see that a patient’s brain physiology and behavioral assessments converg and were clinically relevant to the patient’s presenting symptoms and difficulties. These observations helped me develop a theoretical framework I call the “Brain to Behavior Approach” (17). In this approach, we conceptualize each patient in light of their genetic vulnerabilities and use electrophysiological and behavioral measures that combine nervous system physiology with objective neurocognitive, limbic, and subjective interviews for careful assessment of their brain function. If the results suggest the patient may benefit from pharmacological intervention, we also may employ a medication challenge (18). In a medication challenge, we initially assess the patient’s electrophysiological and behavioral baselines. Then, we administer an acute medication dose and gather a second set of electrophysiological and behavioral data. Although these are time-intensive studies; within just one day, we can learn how the patient responds to a particular medication. . In addition, the overall data allows us to create a comprehensive clinical picture by identifying and prioritizing key converging variables (19). Lastly, we review these data with the patient and, if appropriate, with the family. During the feedback process, the data often enable family members to understand difficult behaviors they assumed were under the patient's volitional control. With this objective information in hand, not only can a clinician begin to address the patient’s current symptoms, but also how the symptoms have impacted familial and peer relationships and how they may impact the patient’s future life trajectory. Subsequently, family and friends come to see the patient as a unique individual with distinctive strengths and weaknesses. Moreover, this information allows the clinician to create a personalized plan for therapy. When deployed in the clinic, the “Brain to Behavior Approach” will not only help patients and their families, but also permit researchers characterize specific symptoms and to address their specific brain-related abnormalities. If adopted, this approach will not only change the clinical paradigm, but will significantly impact how we design future studies and our conceptualization of behavioral disorders.
